# Application and Comparative Study of Generative Artificial Intelligence for Epidemic Prediction of Coronavirus Disease

**DOI:** 10.7759/cureus.91318

**Published:** 2025-08-30

**Authors:** Zongjing Liang, Gongcheng Liang, Yun Kuang, Zhijie Li

**Affiliations:** 1 Artificial Intelligence and Infectious Disease Prediction Models, School of Economics and Management, Guangxi Normal University, Guilin, CHN; 2 Computer Technology Applications and Infectious Disease Prediction Models, Network and Educational Technology Center, Guilin Normal University, Guilin, CHN; 3 Knowledge Engineering and Information Processing, Guilin Normal University, Guilin, CHN

**Keywords:** covid-19, generative ai, model compare, public health, time series forecasting

## Abstract

Background and objectives

In the past twenty years, several large-scale coronavirus outbreaks have caused heavy loss of life and serious economic damage worldwide. Current global surveillance suggests that similar epidemics may occur again, making timely and accurate forecasting an urgent priority. Yet, many existing prediction methods, mainly based on traditional statistical or machine learning techniques, still struggle to deliver both speed and precision. This study explores a generative artificial intelligence-driven approach aimed at narrowing these gaps.

Methods

Nine models (three statistical models, three machine learning models, and three generative artificial intelligence models) were compared using weekly COVID-19 case and death data from the United States (US), the United Kingdom (UK), Germany (GE), and Russia (RU) from March 15, 2020, to April 15, 2023. The statistical models used are simple moving average (SMA), simple exponential smoothing (SES), and the Holt linear trend model (Holt). The machine learning models used are k-nearest neighbor regression (KNN), regression tree (RTree), and multilayer perceptron (MLP). The generative AI models used are ChatGPT, DeepSeek (DS), and Kimi. A custom MATLAB program was used to solve the statistical and machine learning models, and the zero-inference forecasting method was used to solve the generative AI model. According to the stepwise prediction theory, error metrics for one-, two-, and three-step forecasts were calculated: mean absolute percentage error (MAPE), mean absolute error (MAE), and root mean square error (RMSE). The forecasting performance of each model was compared by comparing the one-, two-, and three-step predicting error metrics.

Results

In our analysis, generative AI models consistently delivered the most accurate forecasts. Kimi, in particular, recorded the smallest errors for death predictions and among the lowest for new cases, while DS and ChatGPT also performed well, clearly surpassing the statistical and machine learning approaches in short-term COVID-19 forecasting.

Conclusion

The results of this study demonstrate that generative AI models demonstrate superior predictive accuracy and robustness in epidemic forecasting compared to traditional statistical and machine learning models. This research is innovative in its application of generative AI technology to public health decision-making, demonstrating its robust epidemic forecasting capabilities. Given these proven advantages, public health authorities can integrate generative AI technology into major infectious disease surveillance systems, promote public health data sharing mechanisms, and incorporate generative AI into epidemic intervention and resource allocation. The implementation of these measures will enable governments and regulatory agencies worldwide to use generative AI to enhance early warning capabilities and improve their response to future infectious disease epidemics.

## Introduction

Since the beginning of this century, human society has experienced a series of serious infectious disease outbreaks. The SARS-CoV outbreak in 2003, the swine flu pandemic in 2009, and other socioeconomic and climatic factors are likely to be important driving factors for future infectious disease outbreaks, proving that large-scale epidemics similar to COVID-19 in the future are not accidental [1]. In particular, in the past two decades, three highly pathogenic and deadly coronaviruses have emerged, namely SARS-COV, MERS-COV, and SARS-COV-2. The spread of such infectious diseases has caused a huge socioeconomic burden and posed a serious threat to human health [2]. Compared with SARS and MERS, COVID-19 has spread faster, partly due to increased globalization, and it is clearly pointed out that "inadequate prediction leads to slow public health response" and emphasizes that a prediction mechanism must be established in the future [3].

Studies have shown that there are many reasons for the outbreak and spread of the above-mentioned infectious diseases, including multi-directional spillover from humans to animals, environmental promotion, and live animal markets. For example, the zoonotic spillover of SARS-COV-2 may be transmitted from mink fur to humans [4]. Recent studies have shown that globally, the dual impact of climate change and socioeconomic factors (such as population growth, GDP, and human development index) has led to an increasing trend in infectious disease outbreaks, especially in densely populated and rapidly urbanizing areas, where the risk is significantly accumulated [5]. In addition, land use change can also increase the frequency of zoonotic spillovers, which is the cause of most modern epidemics. Although the true causes of many epidemics remain to be studied, global efforts can improve the ability to prevent and contain outbreaks [6].

In order to effectively respond to major infectious diseases that may re-emerge in the future, it is urgent and necessary to establish a real-time monitoring and early warning system. The current prediction and monitoring systems are still imperfect, and the epidemic response system still needs to be reformed. How to improve response speed and judgment accuracy, and build an early warning system for infectious diseases, is one of the current goals of infectious disease prevention and control [7]. Improving the performance of prediction technology is one of the effective response measures. Prediction can help prepare for possible threats and consequences, so prediction technology plays a very important role in generating accurate predictions [8]. Current prediction technologies are mainly divided into two categories: one is traditional statistical models, such as regression analysis, and the other is machine learning models, such as LSTM models [9]. However, existing prediction methods all have shortcomings, such as large errors and an inability to predict in real time. Therefore, prevention and control agencies should be cautious when formulating public health strategies based on the prediction results of mathematical models [10].

In recent years, the emergence of generative artificial intelligence has provided a new tool for medical research. This tool is real-time and easy to implement, without the need for complex computational programming. Since its birth in November 2022, generative artificial intelligence has been applied in the medical field. For example, ChatGPT is used to predict cancer susceptibility genes, providing a new method for pediatric tumor diagnosis [11]. ChatGPT-4O and ChatGPT-4O Mini were compared with the AHA's established pediatric protocol to help integrate it into the emergency response framework [12]. ChatGPT-4O was applied to medical education and knowledge assessment [13]. Patient education guidelines created by ChatGPT and Google Gemini for acute otitis media, pneumonia, and pharyngitis were compared [14]. The understandability, feasibility, quality, and misinformation of medical information provided by four chatbots were evaluated [15].

In addition to generating content and reasoning tests, generative AI can also be used for prediction. Generative AI prediction models are a new data prediction method, namely, a zero-shot prompt method that can be used for analysis without prior data training. The results of using the zero-shot prompt method for predictive analysis include: the first attempt to predict tourism demand for ChatGPT in various time scenarios [16]. Large language models (LLMs) performed well in various reasoning tasks and demonstrated excellent reasoning ability by applying zero-based chain (CoT) prompts [17].

The zero-shot prompt method is a type of Chain-of-Thought (CoT), which is a technology that reveals and guides LLMs to think and reason step by step [18]. CoT prompts can improve the performance of generative AI in dealing with complex tasks. The zero-shot prompt method can directly let the model solve the problem without any examples. It is a prompt method that does not require examples to guide large models to automatically reason step by step. It is characterized by simple operation and fast deployment.

In the prediction of the COVID-19 epidemic, it is crucial to predict the number of cases and the spread trend in the short term. Such tasks typically require models with excellent nonlinear modeling capabilities and predictive stability. Based on this, this article applies generative AI to epidemic prediction and examines its performance. Incorporating stepwise prediction theory, this article uses nine different models for comparative analysis. These nine models include three traditional statistical models, three machine learning models, and three generative AI models. These statistical models include Holt’s linear trend method (Holt), simple exponential smoothing (SES), and simple moving average (SMA); machine learning models include K-nearest neighbors regression (KNN), multilayer perceptron (MLP), and regression Tree (RTree); and generative AI models include ChatGPT, DeepSeek (DS), and Kimi. The research data covers epidemic data from the United States (US), the United Kingdom (UK), Germany (GE), and Russia (RU), covering the period from March 15, 2020, to May 14, 2023, and includes weekly counts of new COVID-19 cases and deaths. Using a multi-step forecasting approach, we compared the three-step forecast error metrics (mean absolute percentage error (MAPE), mean absolute error (MAE), and root mean square error (RMSE)) of nine models. By comparing these error metrics, we compared the forecast performance of the nine models (three types of models). This study provides a new research paradigm for predicting major infectious diseases in the AI era.

The primary objective of this study is to evaluate and compare the predictive performance of generative artificial intelligence models with traditional statistical and machine learning models in epidemic forecasting. The secondary objectives are: to assess the generalization and robustness of these models across multiple countries and epidemic indicators, to provide quantitative evidence of the advantages of generative AI in epidemic prediction, and to explore the practical implications of integrating generative AI into public health surveillance and decision-making systems.

## Materials and methods

Research data

**Figure 1:**
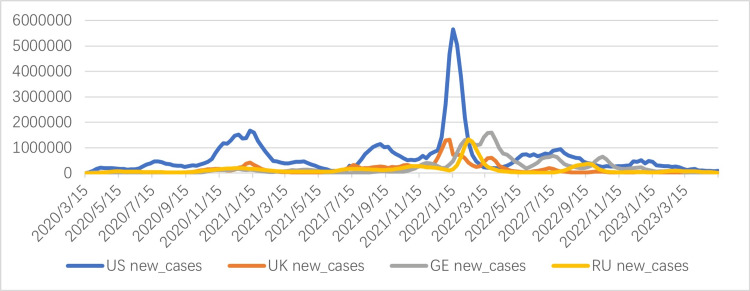
Weekly new COVID-19 infections from March 15, 2020, to May 14, 2023 The horizontal axis represents time, and the vertical axis represents the number of infections. Data source: World Health Organization. US: United States; UK: United Kingdom; GE: Germany; RU: Russia

**Figure 2:**
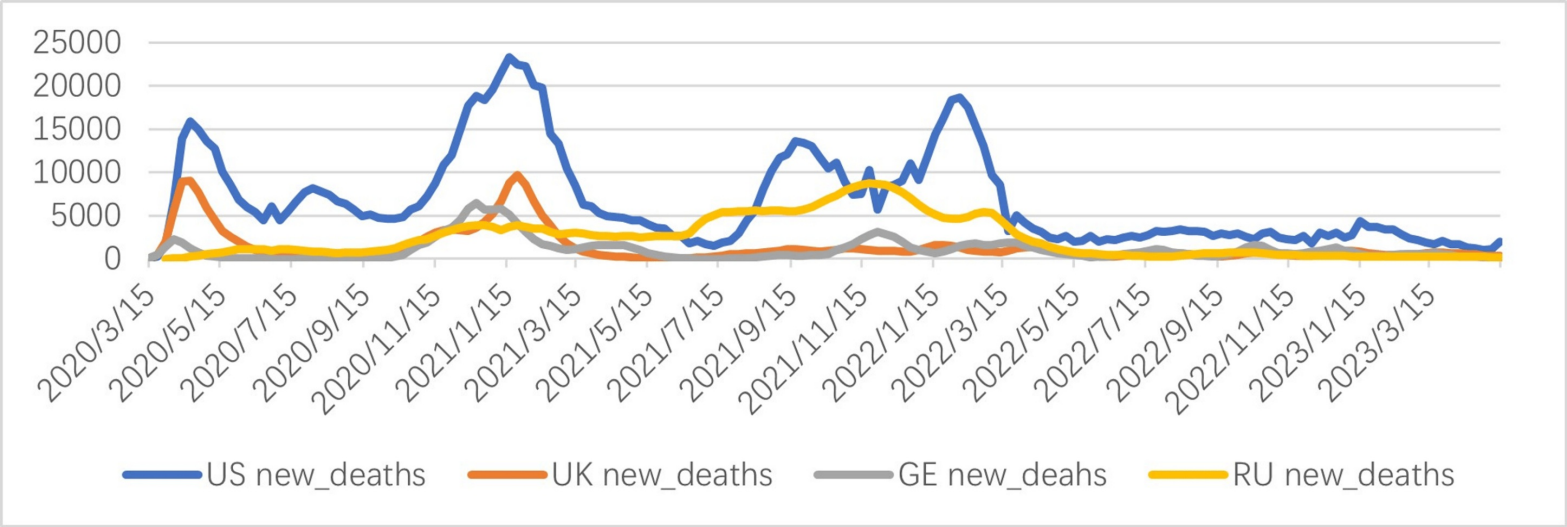
Daily new COVID-19 deaths from March 15, 2022, to April 15, 2023 The horizontal axis represents time, and the vertical axis represents the number of deaths. Data source: World Health Organization. US: United States; UK: United Kingdom; GE: Germany; RU: Russia.

The research data consists of weekly new COVID-19 cases and deaths in the US, the UK, GE, and RU from March 15, 2020, to May 14, 2023. The raw data can be downloaded from the World Health Organization website: https://data.who.int/dashboards/covid19/data?utm_source. Go to the World Health Organization data download page and click "Weekly COVID-19 cases and deaths by date reported to WHO" to download the data file: WHO-COVID-19-global-data.exe. This data file contains data for new cases, cumulative cases, new deaths, and cumulative deaths for countries around the world. For this research, we downloaded new cases and new deaths data for the US, the UK, GE, and RU. The data queuing principle is to capture data from the research period; data from other periods is not included in the calculation. The daily new COVID-19 cases and deaths are shown in Figure 1 and Figure 2, respectively.

Figure 1 shows the changing trends in the daily number of new COVID-19 infections in the US, UK, GE, and RU from March 15, 2020, to May 14, 2023. Analyzing the data fluctuations in the figure, we can draw the following conclusions:

The US epidemic has the greatest volatility. As can be seen from the figure, the US data exhibits cyclical characteristics, with five peaks during this period, the largest of which occurred on January 16, 2022, with a peak of 5650933 cases, indicating the rapid spread of the epidemic. The high number of infections and large peaks may be due to regulatory measures and virus mutations.

The number of new infections in the other three countries, the UK, GE, and RU, remained relatively stable. As the chart shows, the weekly infection data for these three countries exhibited a relatively stable trend. While there were cyclical patterns, the peak growth rate was relatively slow. The fluctuations in the data for these three countries may be related to the emergence of new virus variants, such as the Omicron virus, in 2021 and early 2022, which led to a surge in infection numbers in these three countries.

Overall, the weekly number of new infections exhibits cyclical characteristics. Between March 15, 2020, and May 14, 2023, all four countries experienced multiple peaks in infection numbers, driven by virus mutations, such as the successive emergence of the Alpha, Delta, and Omicron variants, which led to multiple infection peaks.

Figure 2 shows the daily new COVID-19 deaths in the US, the UK, GE, and RU from March 15, 2020, to April 15, 2023. This chart helps analyze the mortality burden and fluctuations in each country during the peak of the epidemic and compares them with the trend of new cases.

The US data shows the highest number. The blue curve in Figure 2 shows significantly higher values throughout the entire time period in four countries, demonstrating a cyclical pattern, with four peaks occurring in April 2020, January 2021, October 2021, and February 2022. This may be due to the toxicity of the virus. Although deaths have decreased with the widespread use of COVID-19 vaccines, deaths due to other personal reasons have continued to peak. While the death toll in the other three countries is relatively small, it continues to show a consistent trend, with no rapid decline.

Research methods

This research method includes the multi-step forecasting strategy, the Holt model, the SES model, the SMA model, and machine learning models such as the KNN model, the MLP model, and the RTree model. The generative AI models ChatGPT, DS, and Kimi are solved using the CoT-prompted forecasting method. A brief description of each of these models is provided below. The three statistical models and three machine learning models in this article are solved using a multi-step forecasting strategy using MATLAB programs. The predictions of the three generative AI models are also based on the CoT-prompted forecasting method, with each AI model directly generating prediction results based on the revealed statements.

Multi-step forecasting strategy theory. This article uses a multi-step time series forecasting method for epidemic research. Multi-step time series forecasting is one of the time series data forecasting methods, which covers a variety of strategies from statistical theory, machine learning, and deep learning. Its methods can be used for one-step-ahead, two-step-ahead, and three-step-ahead forecasting [19].

Stepwise prediction theory is a forecasting paradigm founded by George Box and Gwilym Jenkins in the 1970s. Its core idea is to forecast multiple future time points, sequentially generating forecast values ​​for each point. Subsequent forecasts use the previous forecast value as input, iteratively predicting the value of the next point. In the era of machine learning, stepwise prediction is widely used for iterative multi-step forecasting (Ben Taieb and Atiya, 2016). This involves using the prediction results of one step as the basis for the next, which is then extended to the next step.

In epidemic forecasting, stepwise prediction theory is particularly useful for modeling short- to medium-term trends under uncertain conditions. Previous research has shown that the spread of infectious diseases exhibits autocorrelation, meaning that previous outbreaks have a persistent impact on subsequent outbreaks. Stepwise prediction is particularly important for the prevention and control of major infectious diseases. Through one-, two-, and three-step forecasts, it provides policy references for real-time monitoring of epidemic spread.

This study applies stepwise prediction theory to infectious disease outbreak forecasting, providing real-time short-term forecasts through a three-step process. This three-step forecast can provide early warning for epidemic prevention and control. This mechanism meets the actual needs of infectious disease monitoring and prevention and control, namely, supporting immediate services and planning.

Holt’s Linear Trend Method Model

The Holt model is also known as Holt's linear trend method. This model is a type of exponential smoothing analysis method and consists of two parts: the estimation term and the trend term [20]. The MATLAB program flow of this article is as follows: (1) First, set the working directory and extract the operation data. (2) Set the Holt model smoothing coefficient. In this article, the parameters α=0.8 and β=0.2 are set. (3) Initialize the level term l, trend, and the 1-3 step forecast value vector. (4) Starting from the third time point, recursively update l(t) and b(t) and calculate each step-ahead forecast. (5) Calculate the MAE, MAPE, and RMSE indicators of the one-step-ahead, two-step-ahead, and three-step-ahead step prediction errors. (6) Output the calculation results.

Simple Exponential Smoothing Model

The SES model is an exponential smoothing method based on time series prediction. The principle is to predict data by weighted average historical values [21]. The MATLAB program flow of this article is as follows: (1) Set the program path and data input. (2) Set the smoothing parameter. (3) Use the rolling window for recursive calculation to predict the three-step prediction value. (4) Calculate the error index value of the three steps. (5) Output the calculation results.

Simple Moving Average Model

The SMA model is the most basic time series smoothing method, suitable for short-term prediction. Its principle is to predict future values by averaging historical data with a fixed window size [22]. The MATLAB program flow of this article is as follows: (1) Set the path and read in data. (2) Set the window size, which is three in this article, that is, a three-step prediction. (3) Calculate the three-step prediction value. (4) Calculate the error index and output the result.

K-nearest Neighbors Regression Model

The KNN model is the KNN regression rolling prediction model. This model is a non-parametric supervised learning model. Its principle is to compare and predict the K historical observation points closest to the input sample [23]. The MATLAB program flow of this article is: (1) Set the initial state and read in the data. (2) Set the initial sample and perform rolling prediction. (3) Calculate the one-/two-/three-step prediction values. (4) Output the results.

Multilayer Perceptron Model

The MLP model is a multi-layer perception model. Its principle is to set the weight coefficients through different optimization techniques, identify the optimal number of layers by using genetic algorithms, and then perform prediction analysis through system training [24]. The MATLAB program flow of this article is: (1) System settings. (2) Set the sliding window size to three and set the training set and test set. (3) Train the network. (4) Perform a three-step prediction. (5) Calculate the error index values of the three-step prediction and output the results.

Regression Tree Model

The RTree model is a non-parametric supervised learning method based on a tree structure. Its principle is to divide the features into several regions by using a recursive method, and then perform numerical prediction in each region [25]. The MATLAB program flow of this article is as follows: (1) Initial state setting and data reading. (2) Sliding window. (3) Training the RTree model. (4) Perform one-/two-/three-step rolling prediction. (5) Calculate the error index value of each step and output the result.

Chain-of-Thought Prompt Prediction Method

CoT is the zero-sample prompt method. This prompt prediction method is the calculation method of the generative artificial intelligence prediction in this article. CoT is the zero-sample learning method, that is, based on zero basic data, by asking questions to the LLM chat robot to directly obtain the prediction value [26]. CoT is the zero-sample prompt method. Its principle is to allow generative artificial intelligence to complete a specified task through simple natural language input without providing any examples or intermediate reasoning. If it is used to predict data, the prediction value can be directly obtained. Generative AI Disclosure for this article: Based on the uploaded data, one-step-ahead, two-step-ahead, and three-step-ahead predictions were directly generated for all samples, along with the corresponding error metrics: RMSE, MAE, and MAPE. Output an Excel file. One worksheet contains the actual values and the one-, two-, and three-step predictions, along with the error metrics for each step. The generative AI models used in this article are ChatGPT, DS, and Kimi.

Prediction Error Metric Selection

The error metrics used in this article are MAPE, MAE, and RMSE. The error metrics used for model evaluation are MAPE, MAE, and RMSE. MAPE represents the relative percentage error between the predicted and actual values. MAE represents the mean absolute value of the prediction errors. RMSE averages the squares of the prediction errors and then takes the square root, amplifying the impact of large errors and making it suitable for evaluating high-risk scenarios.

Weighted Average Error Calculation Method

To facilitate comparison of the forecasting performance of different models, this article combines the three-step forecast errors using a weighted average. Because the one-step-ahead step is the most important of the three steps in the three-step forecasting theory, as it is the most critical step for short-term forecasts, this article sets the error weights for the three steps to w₁=0.5, w₂=0.3, and w₃=0.2 (where w represents the weight). For the three error metrics (MAE, MAPE, and RMSE), the three-step errors are weighted averaged as follows: Weighted_Error=w₁·E₁+w₂·E₂+w₃·E₃. The forecasting performance of different models is then compared using Weighted_Error.

Ethics statement

The World Health Organization's (WHO) aggregated COVID-19 surveillance data was made publicly available for this investigation. The dataset is fully anonymized and contains no identifiable personal information. The authors’ institution does not have a dedicated ethics committee for studies using publicly available, non-identifiable data. According to the research content and relevant regulations, this study does not require ethical review.

## Results

Empirical results were obtained using MATLAB programs to calculate various statistical models and machine learning models combined with multi-step forecasting theory. The MATLAB platform used in this article is MATLAB R2022a (MathWorks Inc., Natick, USA). The ChatGPT version used is ChatGPT-40. The DS version used in this article is DS-V3. The Kimi version number is Kimi-K2-0711-preview.

The prediction error metrics of the generative AI models were derived using the CoT zero-shot hinting method. The calculation process first used a self-developed MATLAB program to calculate the one-step-ahead, two-step-ahead, and three-step-ahead errors (MAPE, MAE, and RMSE) for three statistical models and three machine learning models for four countries (the US, the UK, GE, and RU). The average of these three error metrics was then calculated as the error of the statistical and machine learning models. The CoT hinting method was used to calculate the three-step error metrics for the three generative AI models, and the average was calculated as the computational error of the generative AI models. 

Calculation steps

First, each country's raw data (number of new cases or deaths) is read into MATLAB. Three prediction metrics (MAPE, MAE, and RMSE) are then calculated for each country's one-step-ahead, two-step-ahead, and three-step-ahead forecasts. Specific error metric values ​​for each country are shown in the tables. Next, the three error metric values ​​are directly calculated using ChatGPT, DS, and Kimi's zero-sample hint method. Excel (Microsoft, Redmond, WA, USA) then calculates the average of each error metric value. Finally, a MATLAB program is run to generate a heat map of the error metrics.

The model parameter settings for the three statistical models and three machine learning models are as follows: (1) Holt’s linear trend model: level smoothing coefficient (α)=0.8, trend smoothing coefficient (β)=0.2, initial level (l₂)=cases(2), initial trend (b₂)=cases(2)-cases(1); (2) SES: smoothing coefficient (α)=0.8, initial smoothing value (s₁)=cases(1), forecast method: one-step, two-step, and three-step rolling forecasts; (3) SMA: window size (k)=3, forecast method: one-step, two-step, and three-step rolling forecasts; (4) KNN regression: number of neighbors (K)=5 (automatically reduced if the sample size is insufficient), input feature dimension=3 (previous three observations), distance metric=Euclidean distance, forecast method: one-step, two-step, and three-step rolling forecasts. (5) MLP: input feature dimension=3 (previous three observations); architecture: input layer=featureInputLayer(3), fully connected layer with 64 neurons+ReLU, fully connected layer with 32 neurons+ReLU, fully connected layer with 1 neuron (output), followed by a regression layer; optimizer: Adam; MaxEpochs=200; initial learning rate=0.005; forecast method: one-step, two-step, and three-step rolling forecasts. (6) RTree: input feature dimension=3 (previous three observations); algorithm: MATLAB's fitrtree (default parameters); forecast method: one-step, two-step, and three-step rolling forecasts.

Example prompt for generative AI forecasting-ChatGPT (English translation): Based on the uploaded data, directly predict the full-sample one-step-ahead, two-step-ahead, and three-step-ahead forecast values, along with the corresponding error metrics (RMSE, MAE, MAPE, MSE). Output an Excel file with two worksheets: one containing the weekly forecast values, and the other containing the error metrics for the one-step, two-step, and three-step forecasts.

The error indicators, MAPE values ​​of the nine models for the number of new cases per week, are shown in Table 1.

**Table 1:** MAPE values ​​of the nine models for the number of new cases per week US: United States; UK: United Kingdom; GE: Germany; RU: Russia; AV: average values; Holt: Holt’s linear trend method; SES: simple exponential smoothing; SMA: simple moving average; KNN: K-nearest neighbors regression; MLP: multilayer perceptron; RTree: regression tree; DS: DeepSeek; MAPE: mean absolute percentage error

One-step-ahead	US	UK	GE	RU	AV
Holt	23.23742	28.5549	32.27541	25.04458	27.27808
SES	19.12382	25.20326	26.96819	19.00354	22.5747
SMA	29.32302	39.31354	42.53399	30.45081	35.40534
KNN	25.18024	35.22073	39.06548	23.99509	30.86538
MLP	14.65236	14.18602	15.22517	9.014754	13.26958
RTree	22.42385	33.69903	44.01403	23.22692	30.84096
chatGPT	19.12382	21.34109	22.63141	16.88341	19.99493
DS	32.5	33.7	31.2	0.223	24.40575
Kimi	21.34	19.68	18.57	12.45	18.01
Two-step-ahead					
Holt	32.30585	39.30639	46.29887	35.90015	38.45282
SES	33.06929	44.6842	48.38332	33.31733	39.86354
SMA	44.31632	57.78691	64.03428	45.27274	52.85256
KNN	38.72814	57.04436	61.17948	36.8551	48.45177
MLP	26.03569	29.1993	35.70415	24.19559	28.78368
RTree	35.6533	59.06187	69.67955	36.71015	50.27622
chatGPT	33.06929	40.76967	44.05967	31.28553	37.29604
DS	48.2	51.2	48.5	0.261	37.04025
Kimi	22.01	20.31	19.23	14.12	18.9175
Three-step-ahead					
Holt	43.84797	51.97453	62.62648	48.33754	51.69663
SES	48.46884	62.9579	70.13278	48.44152	57.50026
SMA	61.61457	75.38719	86.3156	61.20432	71.13042
KNN	56.12492	80.95036	87.73924	50.73045	68.88624
MLP	37.81689	41.26985	57.69764	45.90628	45.67267
RTree	51.27251	83.79647	94.67415	53.33456	70.76942
chatGPT	48.46884	59.39089	64.8963	45.81412	54.64254
DS	63.7	68.9	62.7	0.298	48.8995
Kimi	22.87	20.95	19.98	15.87	19.9175

**Figure 3:**
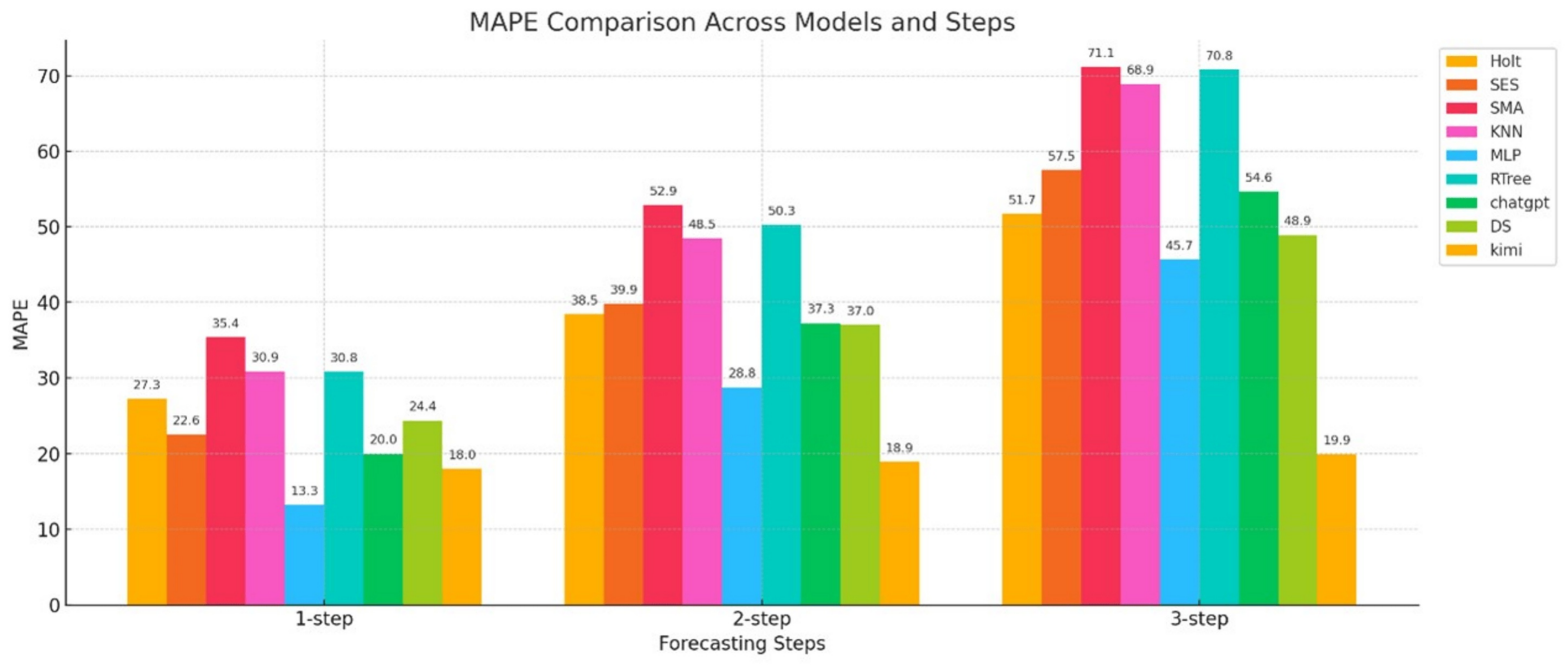
Bar chart of the MAPE error metrics for three-step forecasts for nine models The horizontal axis represents the number of forecast steps (one-step-ahead, two-step-ahead, and three-step-ahead), and the vertical axis represents the MAPE value. Holt: Holt’s linear trend method; SES: simple exponential smoothing; SMA: simple moving average; KNN: K-nearest neighbors regression; MLP: multilayer perceptron; RTree: regression tree; DS: DeepSeek; MAPE: mean absolute percentage error

The MAPE error metrics for the nine models calculated are shown in Figure 3.

Figure 3 shows a bar chart of the MAPE error for three-step forecasts for three types of models. First, we examine the changes in error for the statistical models (Holt, SES, and SMA). As shown in the figure, in the one-step-ahead error comparison, the SES model achieved a MAPE of 22.6, the Holt model achieved a MAPE of 27.3, and the SMA model achieved a MAPE of 35.4, indicating that the SES and Holt models performed well. However, the MAPE values for the two-step-ahead and three-step-ahead models increased rapidly, with the errors becoming increasingly larger. The SMA model achieved an MAPE of 71.1 for the third-step prediction. The MAPE values for the three-step prediction indicate that statistical models perform poorly in predicting the weekly number of new COVID-19 cases, as measured by the MAPE. This may be due to the irregular, volatile, and random nature of the case data.

The next best performers were machine learning models (KNN, MLP, and RTree). In the three-step forecasting, MLP achieved the smallest value and performed the best, with MAPE values of 13.3, 28.8, and 45.7 for one-step, two-step, and three-step predictions, respectively. Although the error also increased, the MAPE values for the first two-step predictions remained relatively low, indicating that this model performs well in short-term forecasting, as measured by the MAPE. The figure also shows that the other two machine learning models performed less well, with relatively large errors.

Finally, the generative AI models (ChatGPT, DS, and Kimi) are presented. Among these models, Kimi's one-step MAPE was 18.0, its two-step MAPE was only 18.9, and its three-step MAPE was only 19.9, showing minimal growth in error. This demonstrates that this model exhibits significant stability and generalization capabilities when predicting epidemic data, as measured by the MAPE error metric. The other two models, ChatGPT and DS, showed a steady increase in MAPE, but their prediction performance was relatively average.

In summary, in the multi-step COVID-19 epidemic prediction task, as measured by the MAPE metric, statistical models maintain acceptable one-step-ahead prediction errors, but their second- and third-step predictions are ineffective. Machine learning models, on the other hand, offer good short-term prediction accuracy but limited stability. Generative AI models, particularly Kimi, demonstrate excellent prediction performance, high accuracy, and strong stability. The other two models perform less well.

The error indicators, MAE values ​​of the nine models for the number of new cases per week, are shown in Table 2.

**Table 2:** MAE values ​​of the nine models for the number of new cases per week US: United States; UK: United Kingdom; GE: Germany; RU: Russia; AV: average values; Holt: Holt’s linear trend method; SES: simple exponential smoothing; SMA: simple moving average; KNN: K-nearest neighbors regression; MLP: multilayer perceptron; RTree: regression tree; DS: DeepSeek

One-step-ahead	US	UK	GE	RU	AV
Holt	148169.7	39005.82	48864.33	32910.65	67237.62
SES	142959.3	39446	50171.22	31778.06	66088.64
SMA	210535.9	56910.9	74382.68	48903.83	97683.32
KNN	190409.2	50282	66614.66	45296.92	88150.69
MLP	82925.03	20188.92	23075.09	10031.08	34055.03
RTree	159026.7	50103.2	63515.07	41366.97	78502.98
chatGPT	142959.3	32713.46	41299.17	26180.23	60788.04
DS	98765	11245	10112	9327	32362.25
Kimi	185230.4	26339.1	29204.6	9876.34	62662.61
Two-step-ahead					
Holt	173379.9	44197.21	58255.19	39991.01	78955.83
SES	241988.4	66069.73	85705.27	56057.29	112455.2
SMA	297313.8	77051.5	103466.5	69895.34	136931.8
KNN	273933.7	72394.21	99983.42	63942.34	127563.4
MLP	190916.3	45235.81	55426.82	28809.86	80097.19
RTree	251820.4	78725.7	99796.55	62240.16	123145.7
chatGPT	241988.4	61108.02	78411.65	50273.43	107945.4
DS	145678	17892	15893	10894	47589.25
Kimi	192104.7	27812.4	31812.4	11234.78	65741.07
Three-step-ahead					
Holt	217400	51737.41	73011.05	49746.65	97973.78
SES	325852.6	84217.36	113552.8	76377.28	150000
SMA	377062.6	89980.98	126398.8	88911.13	170588.4
KNN	357906.8	91195.33	130047.1	81506.76	165164
MLP	322469	69079.72	88379.95	52678.64	133151.8
RTree	345435	98811.29	128172.5	81819.77	163559.6
chatGPT	325852.6	81196.21	107877.8	71946.58	146718.3
DS	198765	24561	20745	12105	64044
Kimi	199876.3	29104.7	33892.7	12345.67	68804.84

**Figure 4:**
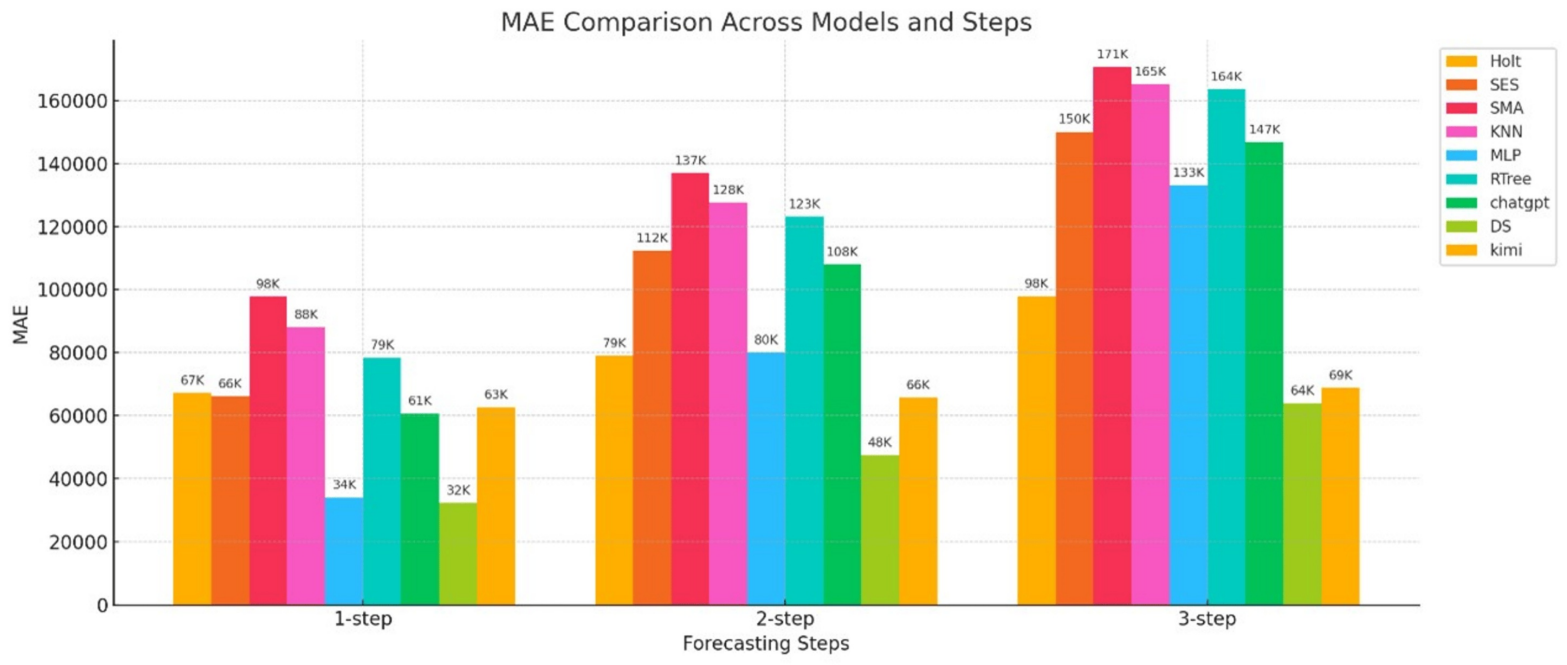
Bar chart of the MAE for three-step forecasts of nine models The horizontal axis represents the number of prediction steps (one-step-ahead, two-step-ahead, and three-step-ahead), and the vertical axis represents the MAE value. MAE: mean absolute error; Holt: Holt’s linear trend method; SES: simple exponential smoothing; SMA: simple moving average; KNN: K-nearest neighbors regression; MLP: multilayer perceptron; RTree: regression tree; DS: DeepSeek

Figure 4 shows the MAE results for the nine models.

Figure 4 shows a comparison of the MAE for three-step forecasts. First, let's examine the statistical models (Holt, SES, and SMA). The MAEs of Holt and SES at one-step are 67K and 66K, respectively, which are not significant. However, as the number of steps increases, the errors rapidly increase. This is especially true for SMA, which increases from 98K at one-step to 171K at three-step, the highest MAE among all models. This indicates that these models exhibit significant distortion in long-term forecasts. The overall MAE error of statistical models is large, and the rate of error growth increases rapidly with increasing number of steps, indicating that these models exhibit significant errors in epidemic data forecasts.

Next are traditional machine learning models (KNN, MLP, and RTree). In comparison, MLP has the smallest MAE value in the one-step-ahead data, but the MAE values of the three machine learning models increase rapidly in the second and third steps. This may be due to the nonlinearity of the original data.

Last among them are generative AI models (ChatGPT, DS, and Kimi). As can be seen from the figure, model DS performs best, with its MAE value increasing from 32K to 48K and 64K. While there is still an upward trend, the increase is small, demonstrating that this model performs well in terms of the MAE error metric. The second-best model is the Kimi model, which also has good stability and a less pronounced trend. The worst MAE error performance is the ChatGPT model, whose MAE value increases rapidly with increasing step number, indicating poor stability.

The error indicators, RMSE values ​​of the nine models for the number of new cases per week, are shown in Table 3.

**Table 3:** RMSE values ​​of the nine models for the number of new cases per week US: United States; UK: United Kingdom; GE: Germany; RU: Russia; AV: average values; Holt: Holt’s linear trend method; SES: simple exponential smoothing; SMA: simple moving average; KNN: K-nearest neighbors regression; MLP: multilayer perceptron; RTree: regression tree; DS: DeepSeek

One-step-ahead	US	UK	GE	RU	AV
Holt	353179	87675.04	90135.05	81689.53	153169.7
SES	342978.5	83758.55	90265.9	79476.1	149119.8
SMA	506973.9	112742.2	132891.7	118870.1	217869.5
KNN	485195.7	115453.4	138640.6	123873.7	215790.8
MLP	189166.4	39554.19	43429.63	17686.23	72459.12
RTree	388830	104169.8	116582.2	103722	178326
chatGPT	342978.5	75433.02	76257.89	66294.1	140240.9
DS	125678	14892	14229	12415	41803.5
Kimi	312847.6	45182.4	51847.2	12034.56	105477.9
Two-step-ahead					
Holt	400290.2	97930.45	103613.2	92739.21	173643.3
SES	581322.3	131218.9	152575.9	136063.9	250295.3
SMA	701043.6	148523.4	183873.1	165605.3	299761.4
KNN	641005.6	151872.1	197026.9	161860.8	287941.3
MLP	571867	91806.21	100341.1	47655.75	202917.5
RTree	616256.6	154063.5	176221.6	149412.6	273988.6
chatGPT	581322.3	126107.4	141731.7	125709.6	243717.8
DS	187654	22765	21876	13882	61544.25
Kimi	324511.2	47263.8	54134.8	14523.12	110108.2
Three-step-ahead					
Holt	476202.9	113314.7	126301.3	110682.4	206625.3
SES	767762.4	162838.9	201445.3	181246.4	328323.2
SMA	847547.3	173646.7	223302.3	201355.7	361463
KNN	728342.3	179573.1	242981.3	190717.6	335403.6
MLP	1039001	145495.5	158211.2	91247.2	358488.8
RTree	792244.4	190857.1	220126.1	189710.9	348234.6
chatGPT	767762.4	159835	193780.4	174086	323865.9
DS	245789	30128	28451	15217	79896.25
Kimi	336892.8	49145.6	56521.4	15987.23	114636.8

**Figure 5:**
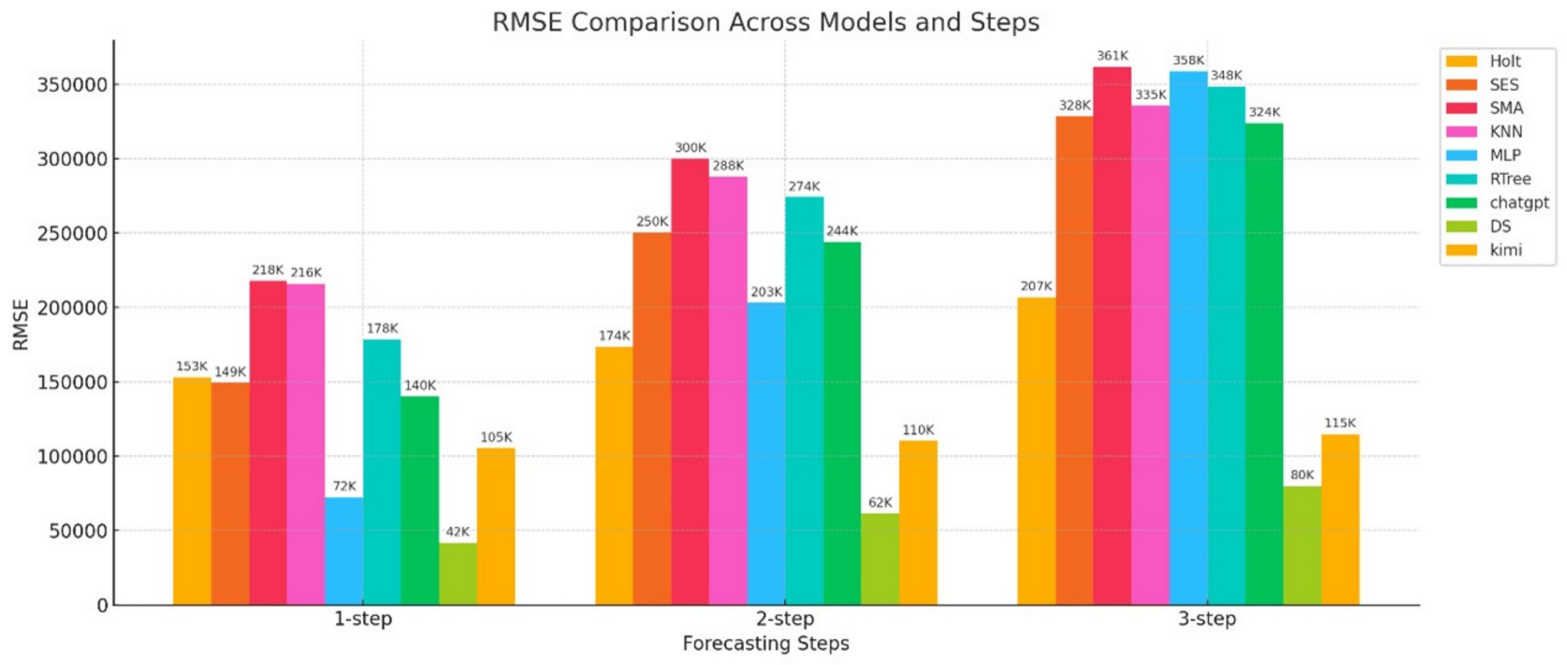
RMSE bar chart for three-step forecasts of nine models The horizontal axis represents the number of prediction steps (one-step-ahead, two-step-ahead, and three-step-ahead), and the vertical axis represents the RMSE value. RMSE: root mean square error; Holt: Holt’s linear trend method; SES: simple exponential smoothing; SMA: simple moving average; KNN: K-nearest neighbors regression; MLP: multilayer perceptron; RTree: regression tree; DS: DeepSeek

The RMSE metrics for the predictions of the nine models are shown in Figure 5.

Figure 5 shows a bar chart of RMSE errors. As can be seen from the figure, when comparing the three models in one-step-ahead prediction, the three statistical models all have larger errors than the machine learning and generative AI models. However, one model each in the machine learning and generative AI models stands out, such as the MLP model and the DS model, whose RMSEs are 72K and 42K, respectively. In two-step-ahead prediction, the RMSE errors of the three statistical and machine learning models increase sharply, indicating that these two models have poor predictive stability. However, the DS and Kimi models, among the generative AI models, perform well, and although their RMSE also increases, the rate of increase is more gradual. In three-step-ahead prediction, the errors of the statistical and machine learning models increase further, but the DS and Kimi models, among the generative AI models, continue to perform well. Therefore, from the perspective of RMSE error, generative AI models have a clear advantage.

To facilitate comparison of the predictive performance of different models, this article uses the weighted average error calculation method to calculate the weighted average (Weighted_Error) of each model. The results are shown in Table 4.

**Table 4:** Weighted averages of the error indicators MAPE, MAE, and RMSE for the nine models US: United States; UK: United Kingdom; GE: Germany; RU: Russia; AV: average values; Holt: Holt’s linear trend method; SES: simple exponential smoothing; SMA: simple moving average; KNN: K-nearest neighbors regression; MLP: multilayer perceptron; RTree: regression tree; DS: DeepSeek; MAPE: mean absolute percentage error; MAE: mean absolute error; RMSE: root mean square error

Model comparison	Holt	SES	SMA	KNN	MLP	RTree	ChatGPT	DS	Kimi
MAPE weighted average	35.5	34.7	47.8	43.7	24.4	44.7	32.1	33.1	18.7
MAE weighted average	76900.3	96780.9	124038.9	115377.2	67687.0	108907.1	92121.3	43266.7	64814.6
RMSE weighted average	170002.9	215313.1	271155.7	261358.5	168802.6	241006.5	208009.0	55344.3	108698.8

**Figure 6:**
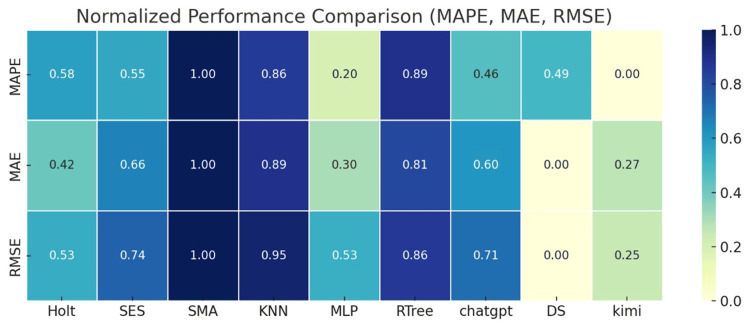
Weighted average normalized potential heatmap of MAPE, MAE, and RMSE for nine models MAPE: mean absolute percentage error; MAE: mean absolute error; RMSE: root mean square error

To visually display the prediction error metrics of different models, the weighted averages of MAPE, MAE, and RMSE are normalized and presented as a heatmap, as shown in Figure 6.

Figure 6 shows a weighted average normalized potential heatmap of MAPE, MAE, and RMSE for nine models. The data in this figure has been normalized. Smaller values indicate smaller errors and better performance, while values closer to one indicate larger errors and poorer performance. The heatmap displays the weighted average of the three error metrics, MAPE, MAE, and RMSE, allowing for horizontal comparison of the predictive performance of different models.

As seen in the heatmap, the statistical models (Holt, SES, and SMA) generally display darker colors, indicating larger errors, with the SMA model exhibiting the highest error. In summary, traditional statistical models cannot reflect the changing trends of the epidemic in a timely manner and can only be used to roughly estimate short-term trends, but are not suitable for accurate short-term or long-term predictions.

The MLP model, one of the machine learning models (KNN, MLP, and RTREE), performs better compared to the statistical models. As can be seen in the figure, the MLP model's error is lighter, indicating that its three error values are smaller, demonstrating good predictive performance. However, the other two models perform poorly. In summary, machine learning models are suitable for general predictive applications, but should only be used as a reference method for addressing the lag issues of sudden epidemics.

Generative AI models, such as DS and Kimi, excel across the board in terms of prediction error. Notably, DS achieves a binomial error of 0.00, the best performance among all models. The Kimi model is second, with three error metrics of 0.00, 0.27, and 0.25, respectively, demonstrating good predictive performance. This demonstrates that generative AI models have excellent short-term and long-term predictive performance and can be used in practical medical prediction applications.

Analysis of error metrics for weekly death predictions using nine models

To comprehensively compare the predictive capabilities of different models for the COVID-19 epidemic and provide reliable forecasting tools for practical use, this study analyzes weekly death counts from four countries (the US, the UK, the GE, AND the RU) between March 15, 2020, and May 14, 2023. The same nine models are applied to conduct a comparative analysis based on their prediction metrics.

The error indicators, MAPE values ​​of the nine models for the number of death counts per week, are shown in Table 5.

**Table 5:** MAPE values ​​of the nine models for the number of death counts per week US: United States; UK: United Kingdom; GE: Germany; RU: Russia; AV: average values; Holt: Holt’s linear trend method; SES: simple exponential smoothing; SMA: simple moving average; KNN: K-nearest neighbors regression; MLP: multilayer perceptron; RTree: regression tree; DS: DeepSeek; MAPE: mean absolute percentage error

One-step-ahead	US	UK	GE	RU	AV
Holt	18.11	37.64	48.40	10.35	28.63
SES	17.15	23.30	31.11	10.39	20.48
SMA	22.87	37.23	50.86	15.63	31.65
KNN	23.48	41.68	57.78	18.05	35.25
MLP	12.99	10.18	15.62	5.55	11.08
RTree	23.45	48.74	78.25	13.62	41.01
chatGPT	16.93	19.65	26.14	9.46	18.04
DS	38.20	36.70	42.10	28.50	36.38
Kimi	14.20	18.70	18.90	12.34	16.04
Two-step-ahead					
Holt	22.62	59.54	74.74	14.78	42.92
SES	24.90	41.80	57.35	17.20	35.31
SMA	31.37	56.73	80.18	22.23	47.63
KNN	31.26	67.10	96.14	25.92	55.11
MLP	17.73	20.99	29.14	10.14	19.50
RTree	31.83	75.04	119.37	20.10	61.58
chatGPT	24.18	38.05	50.70	16.54	32.37
DS	41.10	39.80	45.30	33.80	40.00
Kimi	16.80	21.40	21.30	15.67	18.79
Three-step-ahead					
Holt	29.07	83.46	104.44	20.92	59.47
SES	33.99	61.80	87.76	23.61	51.79
SMA	40.70	77.38	115.38	28.66	65.53
KNN	39.21	96.61	145.40	33.78	78.75
MLP	25.06	35.76	44.08	15.46	30.09
RTree	41.56	117.71	166.05	26.89	88.05
chatGPT	32.96	57.33	79.67	23.06	48.26
DS	43.50	41.20	47.80	38.20	42.68
Kimi	19.50	23.90	23.70	18.92	21.51

**Figure 7:**
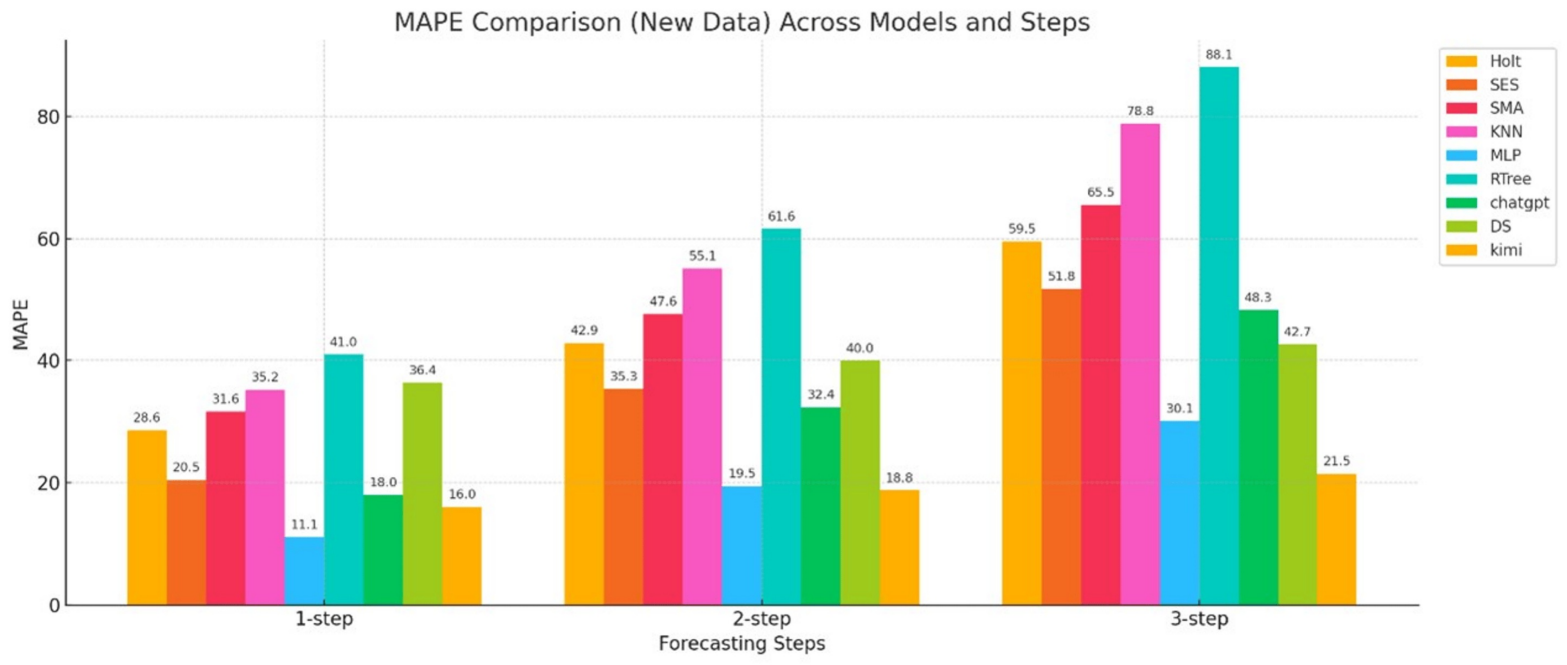
Bar chart of the MAPE values for the three-step prediction of daily deaths for nine models The horizontal axis represents the number of prediction steps, i.e., one-step-ahead, two-step-ahead, and three-step-ahead, and the vertical axis represents the MAPE value. Holt: Holt’s linear trend method; SES: simple exponential smoothing; SMA: simple moving average; KNN: K-nearest neighbors regression; MLP: multilayer perceptron; RTree: regression tree; DS: DeepSeek; MAPE: mean absolute percentage error

**Figure 8:**
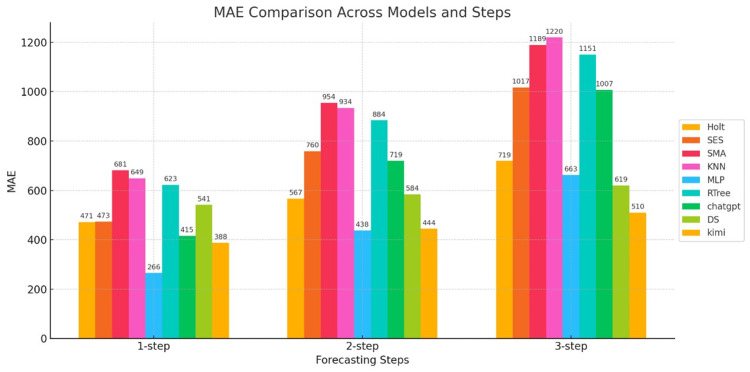
Bar chart of the MAE values for the three-step prediction of daily deaths using nine models The horizontal axis represents the number of prediction steps, i.e., one-step-ahead, two-step-ahead, and three-step-ahead, and the vertical axis represents the MAE value. MAE: mean absolute error; Holt: Holt’s linear trend method; SES: simple exponential smoothing; SMA: simple moving average; KNN: K-nearest neighbors regression; MLP: multilayer perceptron; RTree: regression tree; DS: DeepSeek

**Figure 9:**
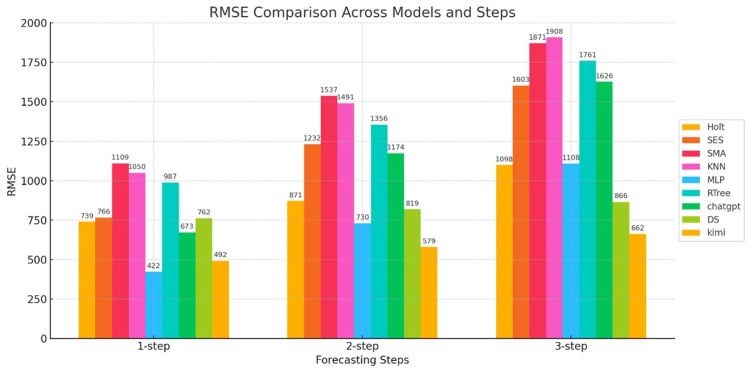
RMSE values for three-step predictions of daily deaths for nine models The horizontal axis represents the number of prediction steps (one-step-ahead, two-step-ahead, and three-step-ahead), and the vertical axis represents the RMSE value. RMSE: root mean square error; Holt: Holt’s linear trend method; SES: simple exponential smoothing; SMA: simple moving average; KNN: K-nearest neighbors regression; MLP: multilayer perceptron; RTree: regression tree; DS: DeepSeek

The calculated error metrics for the nine models, namely MAPE, MAE, and RMSE, are shown in Figures 7, 8, 9.

The error indicators, MAE values ​​of the nine models for the number of death counts per week, are shown in Table 6.

**Table 6:** MAE values ​​of the nine models for the number of death counts per week US: United States; UK: United Kingdom; GE: Germany; RU: Russia; AV: average values; Holt: Holt’s linear trend method; SES: simple exponential smoothing; SMA: simple moving average; KNN: K-nearest neighbors regression; MLP: multilayer perceptron; RTree: regression tree; DS: DeepSeek; MAE: mean absolute error

One-step-ahead	US	UK	GE	RU	AV
Holt	1117.83	350.63	242.18	175.26	471.48
SES	1109.32	344.77	241.43	197.87	473.35
SMA	1548.53	518.43	362.18	294.71	680.96
KNN	1446.31	435.60	325.34	389.25	649.13
MLP	745.54	95.93	112.57	108.00	265.51
RTree	1406.37	429.41	352.75	301.68	622.55
chatGPT	996.67	291.01	209.78	163.91	415.34
DS	1423.00	298.70	245.20	198.20	541.28
Kimi	987.45	298.76	78.20	187.23	387.91
Two-step-ahead					
Holt	1335.02	422.32	278.97	230.28	566.65
SES	1725.60	566.45	407.85	338.55	759.61
SMA	2156.25	724.92	509.09	427.50	954.44
KNN	2029.35	663.84	507.34	534.87	933.85
MLP	1083.83	217.15	235.85	213.53	437.59
RTree	1957.67	640.01	511.81	425.61	883.78
chatGPT	1628.07	557.91	386.87	304.94	719.45
DS	1512.00	329.40	263.70	231.60	584.18
Kimi	1123.67	329.14	89.40	234.56	444.19
Three-step-ahead					
Holt	1683.44	523.45	354.96	314.25	719.03
SES	2303.22	748.18	549.53	466.88	1016.95
SMA	2703.57	858.56	643.06	552.55	1189.43
KNN	2619.36	874.77	712.89	672.10	1219.78
MLP	1567.57	380.56	363.95	338.28	662.59
RTree	2534.65	856.65	669.49	541.73	1150.63
chatGPT	2269.48	784.76	536.48	436.37	1006.77
DS	1587.00	354.20	278.40	258.30	619.48
Kimi	1298.34	352.88	97.80	289.34	509.59

The error indicators, RMSE values ​​of the nine models for the number of death counts per week, are shown in Table 7.

**Table 7:** RMSE values ​​of the nine models for the number of death counts per week US: United States; UK: United Kingdom; GE: Germany; RU: Russia; AV: average values; Holt: Holt’s linear trend method; SES: simple exponential smoothing; SMA: simple moving average; KNN: K-nearest neighbors regression; MLP: multilayer perceptron; RTree: regression tree; DS: DeepSeek; RMSE: root mean square error

One-step-ahead	US	UK	GE	RU	AV
Holt	1613.75	717.25	363.00	263.28	739.32
SES	1676.55	712.71	366.66	308.61	766.13
SMA	2351.42	1068.91	549.65	467.88	1109.47
KNN	2066.08	919.20	624.65	589.60	1049.88
MLP	1147.58	185.21	183.47	172.25	422.13
RTree	1991.90	872.12	632.99	452.45	987.37
chatGPT	1503.98	610.83	319.21	256.18	672.55
DS	1892.00	482.30	387.50	285.70	761.88
Kimi	1234.56	385.42	104.30	245.67	492.49
Two-step-ahead					
Holt	1883.18	834.38	436.70	328.55	870.70
SES	2625.78	1154.14	613.45	533.60	1231.74
SMA	3268.59	1435.13	768.02	676.33	1537.02
KNN	2973.42	1311.26	874.95	805.04	1491.17
MLP	1652.30	517.03	397.40	352.41	729.78
RTree	2734.77	1214.01	831.04	643.46	1355.82
chatGPT	2475.57	1146.33	589.48	484.26	1173.91
DS	2012.00	523.10	412.30	327.40	818.70
Kimi	1456.78	428.91	118.70	312.45	579.21
Three-step-ahead					
Holt	2352.58	1032.34	560.01	449.01	1098.49
SES	3416.55	1425.44	832.49	737.80	1603.07
SMA	4004.85	1645.26	967.03	866.92	1871.02
KNN	3860.11	1607.59	1142.00	1020.80	1907.63
MLP	2341.49	952.16	589.21	548.30	1107.79
RTree	3573.60	1621.44	1016.00	831.09	1760.53
chatGPT	3430.63	1570.55	811.67	691.77	1626.16
DS	2115.00	556.80	433.10	358.10	865.75
Kimi	1678.90	459.62	129.50	378.92	661.74

Combining the three error graphs above, we can conclude the following:

Statistical Models

Figure 7 shows that the MAPE values of the statistical model increase rapidly compared to other models, whether in the first, second, or third-step predictions. This indicates that the model tends to follow a linear trend and cannot reflect the weekly death count and possible nonlinear characteristics. Therefore, it can be concluded that the statistical model lacks practical predictive ability. MAE indicator analysis, Figure 8, shows that the three statistical models lack nonlinear predictive ability, especially the SMA model, which exhibits large prediction accuracy errors. RMSE indicator analysis, Figure 9, shows that the statistical model errors are large and grow rapidly, especially the SMA model, whose RMSE remains at the error threshold, demonstrating its poor predictive ability for weekly death data.

Machine Learning Models

MAPE indicator, Figure 7, shows that although machine learning model prediction methods also have the problem of error accumulation in practical applications, especially error propagation during the three-step prediction process, the RTree model demonstrates excellent predictive ability, indicating good adaptability. MAE indicator, Figure 8, shows that the MLP model performs best among all machine learning models, with small errors in all three-step predictions. In terms of the RMSE metric, the MLP model also performed exceptionally well, demonstrating strong stability in short-, medium-, and long-term forecasts.

Generative AI Models

As shown in Figure 7, the MAPE values of the generative AI models stand out across the first to third prediction steps, outperforming the other three models. Model Kimi, in particular, exhibits very stable MAPE values for short-, medium-, and long-term forecasts, with minimal error accumulation, demonstrating strong predictive power. As shown in Figure 8 for the MAE metric, Kimi is the best-performing model among the three generative AI models. Regarding the RMSE metric, generative AI models show a clear advantage, with Kimi leading, followed by DS and then ChatGPT. However, ChatGPT still outperforms traditional statistical and machine learning models.

Similarly, for the weekly number of new COVID-19 deaths, this study uses a weighted average error method to compute the weighted error (Weighted_Error) for each model, facilitating comparison of their predictive performance. The calculation results are shown in Table 8.

**Table 8:** MAPE, MAE, and RMSE weighted averages of the nine models US: United States; UK: United Kingdom; GE: Germany; RU: Russia; AV: average values; Holt: Holt’s linear trend method; SES: simple exponential smoothing; SMA: simple moving average; KNN: K-nearest neighbors regression; MLP: multilayer perceptron; RTree: regression tree; DS: DeepSeek; MAPE: mean absolute percentage error; MAE: mean absolute error; RMSE: root mean square error

Model comparison	Holt	SES	SMA	KNN	MLP	RTree	chatGPT	DS	Kimi
MAPE weighted average	39.08	31.19	43.22	49.91	17.41	56.59	28.38	38.72	17.96
MAE weighted average	549.54	667.95	864.70	848.67	396.55	806.54	624.86	569.79	429.13
RMSE weighted average	850.57	1073.20	1390.04	1353.82	651.56	1252.54	1013.68	799.70	552.35

**Figure 10:**
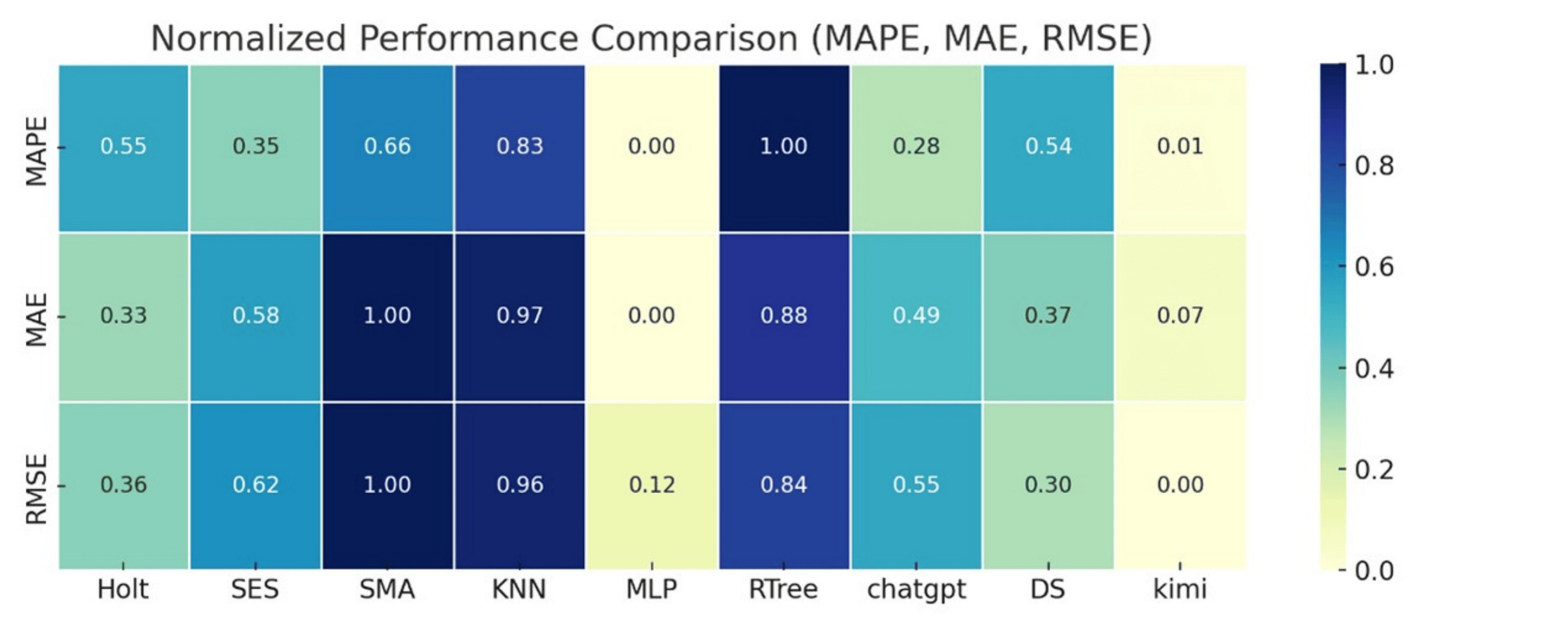
Weighted average normalized potential heatmap of MAPE, MAE, and RMSE for nine models

To visually display the prediction error metrics of different models, the weighted averages of MAPE, MAE, and RMSE are normalized and presented as a heatmap, as shown in Figure 10.

Figure 10 shows a weighted average normalized potential heatmap of MAPE, MAE, and RMSE for nine models, calculated based on the weekly number of new COVID-19 deaths. This heatmap displays the weighted average of the three error metrics, MAPE, MAE, and RMSE, allowing for a horizontal comparison of the predictive performance of different models.

As shown in Figure 10, statistical models (Holt, SES, and SMA), the three statistical models have darker colors in the three error dimensions, indicating larger errors. All three models are generally below average, with SMA performing the worst, indicating that it is not suitable for COVID-19 prediction analysis. As shown in Figure 10, machine learning models (KNN, MLP, and RTree), KNN and RTree have darker colors, indicating large errors, but MLP performs very well, outperforming all models. The generative AI models (DS and Kimi) performed well overall, with Kimi performing particularly well, demonstrating its exceptional predictive capabilities for handling nonlinear data.

Model performance and statistical significance test

We used the paired-sample nonparametric Wilcoxon signed-rank test to examine the forecast errors for the four countries and assessed the statistical significance of the performance differences between the generative AI model Kimi and eight comparison models on six metrics (MAPE, MAE, and RMSE for weekly new cases and weekly new deaths). For weekly new cases, model Kimi achieved a significantly lower MAPE than all models (p<0.05), including MLP and DS, with most comparisons reaching high significance (p<0.001). In terms of MAE, Kimi significantly outperformed SES, SMA, KNN, RTree, ChatGPT, and DS (p<0.01), while the difference from Holt and MLP was not significant. In terms of RMSE, Kimi significantly outperformed all models except MLP and achieved the greatest difference from the statistical model (p<0.001).

For weekly deaths, Kimi's MAPE was significantly lower than that of Holt, SES, SMA, KNN, RTree, ChatGPT, and DS (p<0.01), and not significantly different from MLP (p≈0.91). In terms of MAE, Kimi significantly outperformed all models except MLP (p≈0.91), and slightly outperformed DS (p≈0.027). In terms of RMSE, Kimi significantly outperformed Holt, SES, SMA, KNN, RTree, ChatGPT, and DS (p<0.01), while the difference with MLP was also not significant (p≈0.052). Overall, Kimi's performance was excellent, showing statistically significant advantages over most statistical and machine learning baselines, particularly in terms of MAPE for morbidity and mortality prediction. However, the advantage of DS over MLP is limited to the MAE of new cases, while DS remains competitive, outperforming Kimi in multiple MAE and RMSE comparisons for both cases and deaths. Overall, our tests suggest that Kimi has an advantage in the retrospective benchmark setting, but not universally.

## Discussion

Potential measurement errors in the COVID-19 data used in this article may affect the accuracy of short-term forecasts. While this article uses the official COVID-19 surveillance dataset from the World Health Organization, which is authoritative, these data may be subject to measurement errors due to reporting delays and revisions. Consequently, differences and noise in the data reported by various countries may introduce errors and affect forecast accuracy.

Lack of explicit control for potential confounders may lead to empirical differences in model performance. This study design did not explicitly account for potential confounders, such as vaccination rates, non-pharmaceutical interventions, population prevalence, and other socioeconomic indicators. Differences in these indicators may affect the validity of predictions and the comparison of results across countries.

The generalizability of our findings is limited by our research objectives and data. This study was limited to the COVID-19 pandemic, drawing on historical epidemic data from four countries. Due to time and resource constraints, we were unable to generalize our findings to other infectious disease analyses, thus limiting our conclusions. Future research aims to extend our methods to other infectious disease forecasting, hoping to achieve universal conclusions.

The applicability of stepwise multi-step forecasting methods may depend on the stability of underlying epidemiological and policy conditions. While the stepwise multi-step forecasting paradigm complements traditional epidemiological models (such as SEIR and agent-based frameworks), its purely data-driven nature may affect the robustness of empirical conclusions due to uncertainties such as data changes and policy interventions. This situation needs to be fully considered in forecasting to ensure the reliability of empirical research conclusions.

Although stepwise prediction theory originates from statistical forecasting, it is not inherently in conflict with traditional epidemiological models; rather, they are complementary. For example, the SEIR model is one of the most classic compartmental models in epidemiology. It divides the population into four non-overlapping compartments and uses a set of ordinary differential equations to describe the temporal evolution of the population in each compartment. Agent-based models (ABMs) are a bottom-up simulation framework that breaks down a system into a large number of heterogeneous, interactive agents capable of independent decision-making, allowing macroscopic phenomena to emerge through microscopic rules. Combining stepwise prediction with these models can achieve the fusion of data-driven and epidemiological modeling, thereby enhancing theoretical integration.

Caution is advised when interpreting the results. This study concludes that generative AI models (particularly Kimi) exhibit low error in empirical studies. However, this conclusion is based on an observational study design, which does not account for potential confounding factors and data limitations. Therefore, we clearly distinguish between empirical results directly driven by data and emphasize the speculative nature of the conclusions. The conclusion section is carefully worded to highlight potential uncertainties.

This article presents a comparative analysis of two heatmaps. Figure 6 and Figure 10 compare model error metrics for case and death counts. Comparative analysis of various models and metrics reveals that COVID-19 data exhibits nonlinear characteristics, making it difficult for traditional statistical and machine learning models to accurately capture data trends. A comprehensive comparison of the error metrics in the heatmaps shows that generative AI models (particularly Kimi and DS) consistently outperform the other two models in terms of accuracy and generalization, suggesting that the use of generative AI models for COVID-19 prediction is an optimal approach.

The findings of this study contribute to the development of a generative AI-based epidemic prediction and early warning platform. This study found that generative AI models (ChatGPT, DS, and Kimi) significantly outperformed traditional models on COVID-19 data from multiple countries, ranking first in all three error metrics (MAPE, MAE, and RMSE). Policy implications of this finding include promoting collaboration between government public health departments and AI research institutions to build a unified national AI-driven epidemic prediction system. Incorporate generative AI into the national disease early warning system and conduct epidemic prediction through real-time modeling, multi-task prediction, and trend analysis. Develop AI models for widespread application in grassroots public health institutions to enhance the ability of local disease control departments to respond to emergency epidemics.

Shake the AI-assisted strategy of short-term prediction and emergency mechanism. The results of this article show that the MLP model of machine learning has advantages in short-term prediction. Its policy implications are to use the MLP model to achieve short-term prediction in the early stage of a major infectious disease outbreak, and then combine the prediction results with the short-term epidemic prevention material survey to realize the intelligentization of anti-epidemic logistics, which can greatly improve the epidemic prevention effect. Combine the AI model with the command center and scheduling plan to achieve rapid response in the early stage and gradually build a long-term mechanism that combines expected warning and long-term trend.

From the empirical results of this article, we can see that by comparing the error indicators RMSE, MAE, and MAPE of the nine models, we can see that generative AI models (especially Kimi and DeepSeek) have higher prediction accuracy than traditional statistical models and machine learning models. The conclusions of statistical models are based on historical data, while the conclusions of generative AI models are based on zero-based reasoning without previous data. The generative AI epidemic prediction method in this article provides a new method for infectious disease prediction. In addition, although Kimi and ChatGPT are built on similar LLM architectures, the reason why Kimi performs better in the conclusions of this study may be due to the following reasons: (1) Model fine-tuning strategies for specific fields make the model better adapted to epidemiological prediction tasks. (2) There are differences in the model training corpus, which lead to differences in output. (3) Differences in the inference parameter settings of different models may also lead to differences in the new knowledge produced. (4) The model's strong ability to integrate local data sources can also improve regional predictions. The above reasons can have a potential impact on the prediction results.

Comparison of the application of generative artificial intelligence prediction models in different fields. Wu et al. [16] first introduced the Zero-shot vs. CoT prompt method into tourism prediction research, revealing the significant impact of the prompt method on the prediction effect of ChatGPT. The study emphasized the horizontal evaluation of the short-term prediction ability of the model. This article, drawing on the research methods of this article, applies zero-shot prediction to COVID-19 epidemic forecasting. This is the first time that statistical models, traditional machine learning models, and generative AI models have been compared side-by-side in epidemic forecasting, comprehensively revealing the strengths and weaknesses of each model. This study employs a one-, two-, and three-step rolling prediction strategy, addressing the practical deployment requirements of epidemic monitoring systems. This research expands the application areas of generative AI models.

This article's generative AI research approach differs from existing research. In recent years, generative AI (Generative AI) has been widely used in the medical field. Specific applications include the use of generative AI technology in medical examinations and medical knowledge question-answering, the quality evaluation of human-computer-generated electronic medical records, drug development research using generative AI, and medical education. Existing applications of generative AI in medicine primarily focus on natural language understanding. However, current research rarely applies generative AI technology to in-depth analysis of medical data. Compared to traditional statistical and machine learning models, the limitations of their theoretical foundations and the complexity of their implementation hinder their in-depth application in the medical field. This article introduces a zero-shot CoT (zero-reveal strategy) prediction model based on the CoT prompt strategy, which overcomes the shortcomings of traditional models and enables rapid prediction results and errors, thus providing a new research paradigm for modern medical data analysis.

The innovations of this article are summarized as follows: First, it introduces a methodological innovation, CoT prompting strategy, specifically the Zero-shot CoT (zero-reveal strategy), which enables data processing through simple conversations and facilitates multi-model comparison. Second, the article constructs a unified comparison framework that encompasses statistical models, machine learning models, and various generative AI models (ChatGPT, DeepSeek, and Kimi), enabling cross-model performance evaluation and enhancing the practical value of the research. Finally, the article implements the design of real-world application scenarios. Using one-, two-, and three-step multi-step rolling forecasting methods, combined with multinational COVID-19 data, this study achieves cross-regional assessments, more closely aligned with real-world public health monitoring and intervention design scenarios. Finally, this study further expands on the research objectives. While current AI research in medicine primarily focuses on the micro level, this article extends this focus to macro-epidemic forecasting, opening up a new research direction for generative AI in population health management.

## Conclusions

To address the possible re-emergence of a coronavirus pandemic, this study examined a forecasting approach that incorporates generative AI. We compared it with more conventional statistical and machine learning methods, aiming to identify techniques that are both accurate and timely for epidemic prediction. The models assessed covered three categories: statistical (SMA, SES, Holt Linear Trend Model), machine learning (KNN, RTree, MLP), and generative AI (ChatGPT, DK, Kimi). Model performance was evaluated using MAPE, MAE, and RMSE. Across the data analyzed, the generative AI models tended to perform better in short-term forecasting than the other model families. Kimi, in particular, recorded the lowest MAPE and RMSE in predicting deaths and also produced relatively small errors for new case forecasts, indicating strong performance within this dataset. DK and ChatGPT also returned low prediction errors, giving them a comparative edge over most of the statistical and machine learning models. Of the latter group, only the MLP model showed consistently competitive short-term results, while the others produced larger deviations. These outcomes are based on descriptive comparisons, and not all differences were tested for statistical significance, which limits the strength of any causal interpretation. In addition, this article also conducted model performance and statistical significance tests. To verify whether the performance differences between models are significant, this article uses weekly data on new cases and deaths from four countries (March 2020 to April 2023). The generative AI model Kimi achieves significantly lower prediction errors than most statistical and machine learning models on six evaluation metrics (paired-sample Wilcoxon signed-rank test, p<0.05), with most differences reaching p<0.001. Its advantage over the MLP is limited to the MAE of new cases, while DS outperforms Kimi in multiple MAE and RMSE comparisons. These results from a retrospective benchmark study indicate that Kimi performs well, but not universally, and further validation of Kimi's performance is necessary in more complex forecasting environments.

Taken together, the findings point to the potential value of generative AI in infectious disease forecasting. Such tools, if their advantages are confirmed in further studies, could contribute to more timely and precise epidemic predictions. That said, the observational nature of the work, the possibility of unmeasured confounding factors, and the focus on data from a specific pandemic period and a limited set of countries mean that the conclusions should be viewed with caution. Broader applications to other diseases and settings will require additional empirical verification.
